# Amniotic band syndrome: Insights from first documented case report in Somalia's low-resource setting

**DOI:** 10.1016/j.ijscr.2025.111135

**Published:** 2025-03-11

**Authors:** Salah Haji Hassan Muhumad, Abdirahman Omer Ali, Hassan Elmi Moumin, Ahmed Abdi Aw Egge, Roukia Mahamad Nour, Mohamed Abubakar Muhumed

**Affiliations:** a College of Health Sciences, School of Medicine and Surgery, Amoud University, Borama, Somalia; b Director of Caafi Hospital, Borama, Somalia; c Borama Regional Hospital, Surgery Department, Borama, Somalia

**Keywords:** Amniotic band syndrome, Congenital anomaly, Prenatal diagnosis, Somalia, Case report

## Abstract

**Introduction:**

Amniotic Band Syndrome (ABS) is a rare congenital anomaly resulting from the entanglement of fetal body parts in ruptured amniotic bands, leading to structural abnormalities. It is associated with significant stillbirth rates and presents various manifestations affecting limbs and other regions. This report documents the first case of ABS in Somalia.

**Case presentation:**

A term neonate was delivered vaginally at 37 weeks and 2 days to a 28-year-old woman with a history of preterm premature rupture of membranes (PPROM). The infant presented with a constriction ring on the right forearm and bilateral clubfoot, but normal limb function and perfusion. Antenatal ultrasounds indicated normal development and moderate oligohydramnios; Doppler ultrasound ruled out vascular compromise.

**Discussion:**

The diagnosis of ABS was made postnatally, revealing gaps in prenatal detection capabilities. Conservative management, including local wound care, resulted in satisfactory outcomes. The etiology supports the “extrinsic theory,” linking limb malformations to amniotic rupture. This case highlights the need for improved prenatal screening and training for healthcare providers in Somalia to enhance early detection of congenital anomalies.

**Conclusion:**

This case underscores the importance of recognizing ABS and improving healthcare practices for congenital anomaly management in resource-limited settings.

## Highlights

This case study underscores the rare occurrence of Amniotic Band Syndrome (ABS), a congenital anomaly resulting from the entanglement of fetal body parts in ruptured amniotic bands, leading to various structural abnormalities. Key highlights include:

• **First Documented Case:** This is the first reported instance of Amniotic Band Syndrome (ABS) in Somalia, expanding the geographical understanding of the condition.
• **Clinical Presentation:** A term neonate delivered at 37 weeks exhibited a constriction ring on the right forearm and bilateral clubfoot, but maintained normal limb function and perfusion.
• **Prenatal History:** The mother had a history of preterm premature rupture of membranes (PPROM) at 34 weeks, which may have contributed to the development of ABS.
• **Diagnostic Findings:** Antenatal ultrasounds indicated normal fetal development with moderate oligohydramnios, and Doppler ultrasound confirmed adequate blood flow.
• **Management Approach:** Conservative management was employed, focusing on local wound care and monitoring, leading to satisfactory outcomes without the need for surgical intervention.
• **Implications for Practice:** The case highlights significant gaps in prenatal detection capabilities and emphasizes the need for enhanced training and awareness among healthcare providers in resource-limited settings to improve early detection of congenital anomalies.

## 1 Introduction

Amniotic band syndrome (ABS) with complex etiology is a rare and serious congenital anomaly of the fetus and appendages, in which the fetal body parts are tangled or wrapped by ruptured amniotic bands, resulting in fetal structural abnormalities and dysfunctions [1]. Amniotic band sequence (ABS) is a constellation of complex congenital anomalies that can be seen in infants without any known genetic mutations. It is said to be responsible for 1/70 stillbirths [2]. Amniotic band syndrome (ABS), constriction band syndrome, or Streeter dysplasia occur sporadically, and its estimated prevalence varies widely from 1 in 1200 to 1 in 15,000 live births [3]. The amniotic band syndrome is a group of disorders involving the limbs, the craniofacial region and the thoraco-abdominal area, with a constricting skin band formed by fibrous tissue of chorioamniotic origin, enveloping the limbs, the body wall and/or the viscera, as the main feature. The origin of these malformations is multifactorial [4]. The origin of these malformations is multifactorial. Early and accurate prenatal diagnosis is the key element of management. The prognosis depends on the severity and location of the malformation. Medical abortion may be proposed in case of severe malformations [4]. This case report marks the first documented case of ABS in Somalia after a comprehensive review of the medical literature and available databases (PubMed, Scopus, Google Scholar, and African Journals Online). Somalia faces significant challenges in healthcare infrastructure, access to specialized medical services, and trained healthcare personnel, particularly in maternal and child health. These factors can significantly impact the diagnosis and management of congenital anomalies like ABS [5,6].This case report marks the first documented case of ABS in Somalia. This case is presented in accordance with the SCARE 2023 guidelines [7].

## 2 Case presentation

**Fig. 1:**
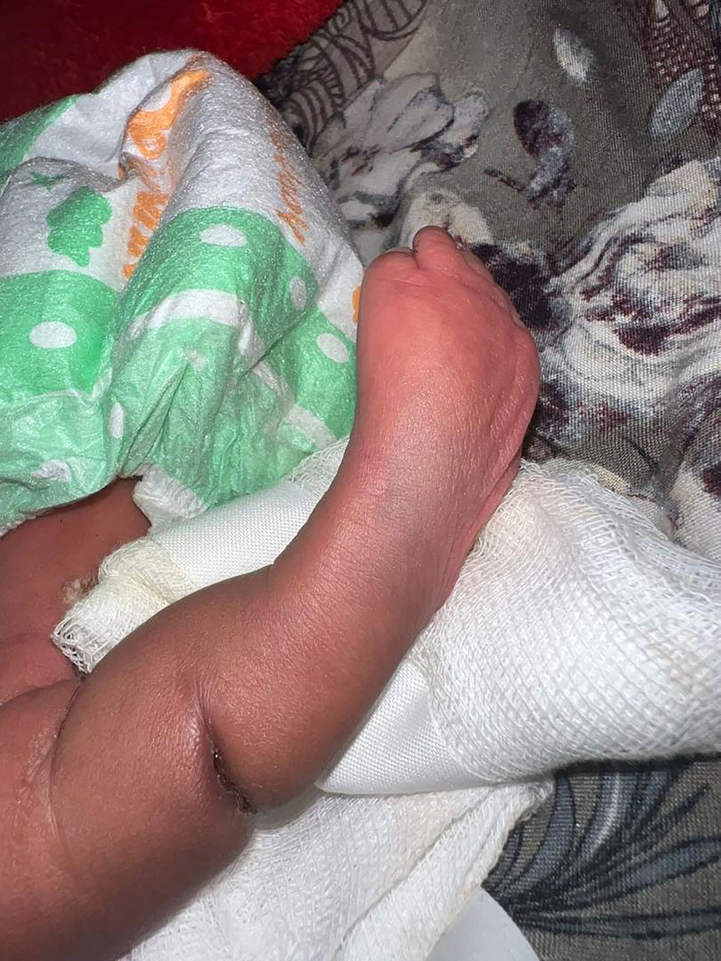
The image shows a neonate's leg with Amniotic Band Syndrome, featuring a constriction ring and swelling.

**Fig. 2:**
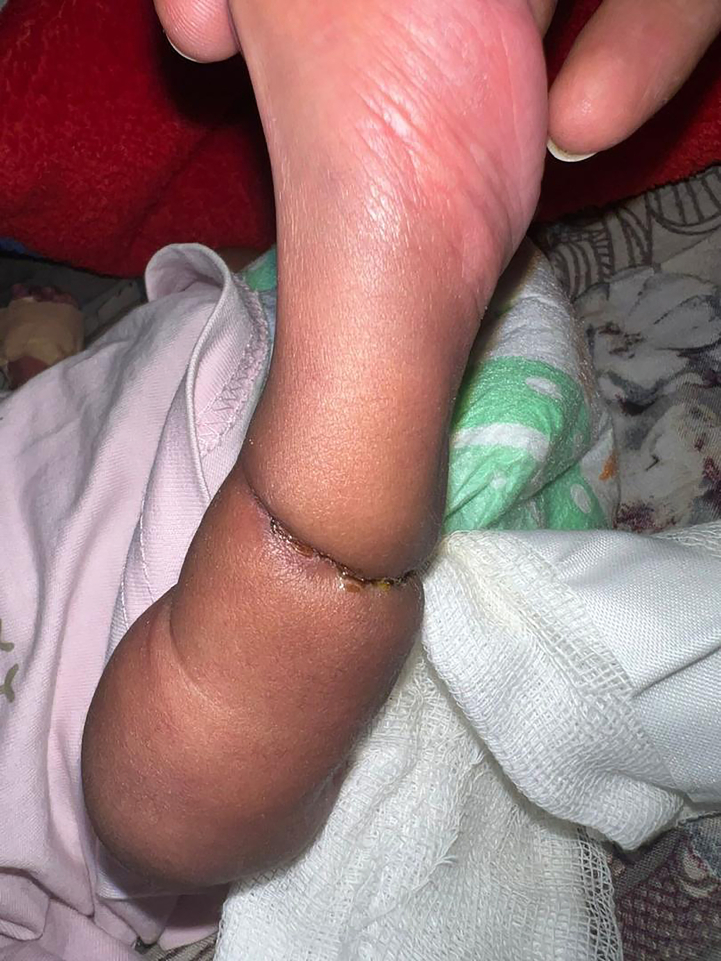
The image shows a neonate's leg with Amniotic Band Syndrome, featuring a constricting band and clubfoot deformity.

A late preterm neonate was delivered vaginally at 37 weeks and 2 days of gestation to a 28-year-old woman, gravida 2, para 1 (G2P1), with a history of preterm premature rupture of membranes (PPROM) at 34 weeks of gestation. Upon delivery, the neonate presented with a constriction ring encircling middle portion of the neonate's leg look Fig. 1**,** Fig. 2**,** resulting in distal swelling while maintaining adequate perfusion. Notably, the infant demonstrated normal movements and reflexes in the affected limb, and physical examination revealed no additional congenital anomalies except of club foot bilaterally.

### 2.1 Clinical findings

Antenatal ultrasonographic assessments revealed fetal growth that aligns with the specified gestational age (GA), with the fetus positioned in a cephalic orientation and exhibiting no gross abnormalities. Furthermore, a moderate degree of oligohydramnios was noted; however, no additional anomalies were detected. Doppler ultrasound confirmed adequate distal blood flow, effectively ruling out the possibility of vascular compromise.

## 3 Therapeutic intervention

The management approach for the neonate was conservative, focusing on local wound care and vigilant monitoring for any signs of ischemia or neurovascular compromise. A collaborative effort involving a multidisciplinary team, consisting of neonatologists, plastic surgeons, and physiotherapists, was undertaken to formulate an all-encompassing management plan. Although the possibility of surgical intervention was considered, it was ultimately postponed due to the lack of any functional impairment.

## 4 Follow-up and outcome

During follow-up, the neonate exhibited satisfactory distal limb function alongside normal growth and developmental milestones. The constriction band was closely monitored, with a plan for potential surgical release should future complications arise, including impaired circulation or restricted movement.

## 5 Discussion

This case report presents a unique case of amniotic band syndrome (ABS) in a late preterm neonate in Somalia, marking the first documented case within the region. The neonate presented with a constriction ring around the middle portion of the neonate's leg, distal swelling, and bilateral clubfoot. The diagnosis was made postnatally, highlighting a crucial gap in prenatal detection in our setting. The conservative management approach adopted focused on local wound care and vigilant monitoring, ultimately resulting in satisfactory distal limb function and normal developmental milestones.

The clinical presentation observed in our case aligns with the heterogeneous manifestations of ABS, as reported in various studies [2,3]. Our patient exhibited limb constriction, a common finding in ABS, consistent with the fibrotic bands causing compression described by Kawamura and Chung [8]. While the severity of ABS can range from minor digit amputations to life-threatening anomalies [9], the presentation in our case was moderate, with adequate limb perfusion and no neurovascular compromise, which was confirmed by Doppler ultrasound, therefore ruling out the possibility of vascular compromise. This finding is similar to the case described by Lamrissi [4]. where an isolated limb malformation was observed. Unlike cases of craniofacial and visceral malformations which may warrant medical abortion [4], our patient had isolated limb malformations that did not impede limb function. The absence of severe deformities allowed for a conservative management approach, similar to that described in other reports [10]. However, many cases have varying degrees of severity, and the long-term outcomes for the patients depend on the severity of the presentation [3,9].

A significant contradiction between our case and some literature lies in the timing of diagnosis. While several studies emphasize prenatal diagnosis via ultrasound from the first trimester [11], our case was identified only postnatally. This highlights a potential gap in prenatal screening practices in Somalia and could point to a limitation in access to high resolution prenatal imaging or specialized diagnostic services. Additionally, moderate oligohydramnios was noted during the antenatal ultrasound, which, in retrospect, could have been an indication of amniotic fluid loss related to early rupture of membranes as suggested previous study [12]. However, this sign was not recognized during the pregnancy, emphasizing a need for increased awareness of atypical ultrasound findings among healthcare providers.

The lack of prenatal diagnosis in this case is particularly concerning given the challenges faced by pregnant women in Somalia. Access to prenatal care is limited by factors such as geographical barriers, poverty, lack of awareness, and insecurity [[13], [14], [15]]. Even when available, prenatal ultrasound services may be basic and lack the resolution necessary to detect subtle anomalies like amniotic bands. Furthermore, there is a shortage of trained sonographers and radiologists capable of interpreting these images accurately [15,16].

Culturally, there may be a reluctance to seek prenatal care due to traditional beliefs or a lack of understanding of its importance. Socioeconomic factors, such as poverty and lack of education, can also contribute to delayed or inadequate prenatal care [[17], [18], [19], [20]]. The ongoing humanitarian crisis and internal displacement in Somalia further exacerbate these challenges, disrupting healthcare services and increasing the vulnerability of pregnant women and their children [[21], [22], [23]]. Even with a prenatal diagnosis, the options for management are limited in Somalia. Access to specialized surgical interventions, such as those that may be required for severe ABS cases, is scarce. Genetic counseling services are virtually non-existent, making it difficult for families to understand the condition and its potential recurrence risk.

The etiology of ABS is still debated. Our case report adds to the existing literature supporting the “extrinsic theory” proposed by Torpin [12] which associated defects of the extremities or limb amputations with amniotic rupture and compression of the bands. The patient had a history of preterm premature rupture of membranes (PPROM) at 34 weeks of gestation which may support the extrinsic theory. In our case, the history of PPROM at 34 weeks gestation strongly supports the extrinsic theory, suggesting that the rupture of the amniotic membranes led to the formation of amniotic bands and subsequent compression of the fetal limb. The current consensus favors the extrinsic theory that involve premature rupture of the membranes with the disruption of fetal parts due to compression by the amniotic bands. [8,10]. However, other theories have been suggested to explain the etiopathogenesis such as the intrinsic theory proposed by Streeter in 1930, which establishes that congenital constriction is a germplasm defect with vascular disruption and change of morphogenesis during gastrulation, forming the fibrous bands and causing limb necrosis [24,25]. While the extrinsic theory is more compelling in this particular case given the PPROM, the possibility of intrinsic factors cannot be entirely ruled out. Subclinical vascular disruption or subtle genetic predispositions could have made the fetus more vulnerable to the effects of amniotic bands. It is possible that both intrinsic and extrinsic factors may have contributed to the development of ABS.

The primary contribution of this case report is the documentation of the first instance of ABS in Somalia, expanding the known geographical distribution of the syndrome. Furthermore, it underscores the importance of recognizing and managing this rare condition, specifically within resource-constrained settings. The successful conservative management of our case highlights the potential for positive outcomes even in the absence of advanced interventions. The lack of prenatal diagnosis in our case is concerning and points to gaps that need to be addressed at policy levels. This study underscores the necessity of strengthening prenatal screening protocols and training for health personnel, especially in low-resource settings such as ours.

Moving forward, there is a crucial need for increased investment in maternal and child health services in Somalia. This includes strengthening prenatal care programs, improving access to diagnostic imaging, training healthcare professionals in the early detection and management of congenital anomalies, and raising awareness among communities about the importance of prenatal care and early intervention [30,31]. We specifically recommend enhancing ultrasound training programs for healthcare providers, implementing dedicated fetal anomaly screening programs, and establishing clear referral pathways for specialized care of congenital anomalies. Further research is also needed to understand the prevalence and risk factors for congenital anomalies in Somalia and to develop culturally appropriate strategies for prevention and management**.**

## 6 Conclusion

This case report highlights the first documented instance of amniotic band syndrome (ABS) in Somalia, emphasizing the critical need for improved prenatal diagnosis in resource-limited settings. The successful conservative management of the neonate illustrates that positive outcomes are possible despite initial diagnostic gaps. While this case may not significantly advance the general understanding of ABS pathophysiology, it provides crucial insights into the challenges of managing this condition within a severely under-resourced environment like Somalia. Our findings underscore the importance of enhancing prenatal screening protocols, investing in healthcare professional training, and establishing clear referral pathways to better address congenital anomalies and improve neonatal health outcomes in similar contexts. Furthermore, this report serves as a call to action for increased global health efforts focused on strengthening maternal and child health services in underserved regions.

## Author contribution

Dr. Salah Haji Hassan Muhamad, Dr. Ahmed Abdi Aw Egge, Dr. abdirahman Omer Ali and Hassan Elmi Moumin individuals contributed to taking history and providing care to the patient throughout her hospital stay. Additionally, Dr. abdirahman Omer Ali, Hassan Elmi Moumin and Dr. Ahmed Abdi Aw Egge contributed to the development of the manuscript.

## Consent

Written informed consent was obtained from the patient's parents/legal guardian for publication and any accompanying images. A copy of the written consent is available for review by the Editor-in-Chief of this journal on request.

## Ethical approval

The study protocol, case investigation, and consent form were thoroughly examined by the institutional review board of the College of Health Sciences at Amoud University. They granted approval for the study, along with the Ministry of Health and Borama Hospital in Awdal Region, Somaliland (BRH-190/2024). Prior to participation, written informed consent was obtained from every individual involved.

## Guarantor

Abdirahman Omer Ali, on behalf of all authors, accept full responsibility for the work.

## Funding

The study did not receive funding.

## Registration of research studies

Not applicable.

## Declaration of competing interest

The authors affirm that there are no conflicts of interest pertaining to the publication of this article.

## References

[1] Niu Z., Meng H., Zhang X., Ouyang Y., Zhang Y., Wu X. (2019). Two case reports: early detection of amniotic band syndrome by adhesion between hand and umbilical cord at 11 to 14 weeks’ gestation. Medicine (Baltimore).

[2] Kathleen Munisteri M., Villazana-Kretzer D., Gonzalez Brown V. (2022). Amniotic band syndrome: a case series. J. Case Reports Images Obstet. Gynecol..

[3] Phan T.H., Nguyen P.T.T., Nguyen P.N., Pham H.H., Ngo Q.D., La Nguyen P.T. (2023). Amniotic band syndrome leading to severe malformations of the newborn: a case report at Tu Du Hospital, Vietnam, and literature review. Ann. Med. Surg..

[4] A. Lamrissi, M. Mourabbih, O. Ouajih, M. Jalal, K. Fichtali, and S. Bouhya, “Amniotic band syndrome: A case report,” *Int. J. Surg. Case Rep.*, vol. 95, no. December 2021, p. 107096, 2022, doi:10.1016/j.ijscr.2022.107096. PMC9112021 35567876

[5] S. M. Hidig, M. Hanfi, and A. Ali, “The challenges facing the healthcare system in Somalia : A review and The Way Forward,” no. February, 2024, doi:10.58614/hi312.

[6] SHDS, “and Demographic Survey 2020,” *SHD Surv.* 2020 *Somalia*, p. 2020, 2020.

[7] Sohrabi C., Mathew G., Maria N., Kerwan A., Franchi T., Agha R.A. (2023). The SCARE 2023 guideline: updating consensus surgical CAse REport (SCARE) guidelines. Int. J. Surg..

[8] Kawamura K., Chung K.C. (2009). Constriction band syndrome. Hand Clin..

[9] Walter J.H., Goss L.R., Lazzara A.T. (1998). Amniotic band syndrome. J. Foot Ankle Surg..

[10] Light T.R., Ogden J.A. (1994). Congenital constriction band syndrome pathophysiology and treatment. Yale J. Biol. Med..

[11] Mrezguia C. (2022). Amniotic band disease: a case report. PAMJ Clin. Med..

[12] Torpin R. (1971). Amnion rupture during gestation and associated fetal malformations. Bull. Am. Coll. Nurse-Midwives.

[13] Haji O.A. (2022).

[14] Miikkulainen A. (2023). Antenatal care utilization and its associated factors in Somalia: a cross-sectional study. BMC Pregnancy Childbirth.

[15] M. Abdillahi, A. Abdirahman, O. Ali, A. Hassan, A. Omer, and A. Farih, “Prevalence and determinants of non - communicable diseases among child - bearing women in Somaliland from a 2020 nationwide survey in Somaliland,” Discov. Public Heal., 2024, doi:10.1186/s12982-024-00371-y.

[16] Kumar S. (2008).

[17] Shirazi Nejad S., Roshan M., Jafarpishe M.S., Hashemi P., Shahsavan M., Shahsavan M. (2024). Prenatal ultrasound detection of fetal amniotic band ingestion in monochorionic diamniotic twin pregnancy: a rare case report. BMC Pregnancy Childbirth.

[18] Ali A.O., Said A.I., Abdilahi M.A., Deheye A.S., Muse A.H. (2024). Prune belly syndrome in a neonate with severe bilateral hydronephrosis: a rare case report from Somalia. Int. J. Surg. Case Rep..

[19] Moumin H.E., Ali A.O., Egge A.A.A., Abdi M.H., Moumin A.E., Muse A.H. (2025). Spontaneous splenic rupture: a rare complication of concurrent malaria and dengue infections – a case report. Int. J. Surg. Case Rep..

[20] Aynalem B.Y., Melesse M.F., Bitewa Y.B. (2023). Cultural beliefs and traditional practices during pregnancy, child birth, and the postpartum period in east Gojjam zone, Northwest Ethiopia: a qualitative study. Women’s Heal. Reports.

[21] et al., “Adherencia a la atención prenatal en el contexto sociocultural de países subdesarrollados: una revisión narrativa,” Horiz. Médico, vol. 23, no. 4, p. e2252, 2023, doi:10.24265/horizmed.2023.v23n4.12.

[22] Grand-Guillaume-Perrenoud J.A., Origlia P., Cignacco E. (2022). Barriers and facilitators of maternal healthcare utilisation in the perinatal period among women with social disadvantage: a theory-guided systematic review. Midwifery.

[23] Heaman M.I. (2015). Barriers and facilitators related to use of prenatal care by inner-city women: perceptions of health care providers. BMC Pregnancy Childbirth.

[24] Said A.I., Abdulahi M., Ali A.O., Koshin A.M., Walhad S.A., Muse A.H. (2024). Left temporal epilepsy unmasking tuberculoma in an immunocompetent adolescent: case report. Atención Primaria Práctica.

[25] H. Situation, “Somalia,” no. 11, pp. 1–9, 2024.

[30] I. Rmncah, “Policy brief Time to act – Making motherhood and childhood safer in Somalia,” no. 3, pp. 1–12, 2023.

[31] A. Health, “Reproductive-Maternal-Neonatal-Child-And-Adolescent-Health-Strategy-2019–2023 (2),” 2019.

